# Negative Influences of the 4th Industrial Revolution on the Workplace: Towards a Theoretical Model of Entropic Citizen Behavior in Toxic Organizations

**DOI:** 10.3390/ijerph16152670

**Published:** 2019-07-25

**Authors:** David. A. L. Coldwell

**Affiliations:** School of Economic and Business Sciences, University of the Witwatersrand, Johannesburg 2050, South Africa; david.coldwell@wits.ac.za; Tel.: +27-011-717-0000

**Keywords:** 4th industrial revolution, workplace, entropic citizenship behavior, toxic organizations

## Abstract

The 4th industrial revolution, referred to as a ‘second coming’ of the ‘digital era,’ has introduced both positive and negative effects on the workplace. While digitalization and automation have taken the drudgery out of work for some and released them to enjoy qualitative improvements at work and higher salaries, others have been thrust into low-paying work and unemployment with negative effects on their well-being and mental health. In many cases stress and threats of job loss created by digital era automation have generated negative workplace behavior and workplace outcomes. The 4th industrial revolution and its burgeoning information technology have presented widespread access to information to stakeholders and the general public about organizational business and environmental performance. This open access to information has driven toxic business leaders to maintain company profitability and environmental sustainability by pressuring employees to find solutions to difficult organizational problems with short timelines attached. Employees often are required to ‘go the extra mile’ to achieve organizational goals through forms of organizational citizenship behavior. Additionally, although organizational citizenship behavior can generate significant benefits for a company, toxic and entropic workplace outcomes can also occur from its more extreme manifestations arising from the stressful circumstances digitalization and automation of work have created. The methodological approach adopted in this paper is a secondary data analysis which uses reliable and valid sources of report documentation to corroborate a theoretical model of organizational citizenship behavior entropy. The theoretical model suggests that extreme forms of organizational citizenship behavior associated with the digital era can create toxic leaders and business organizations that lead to organizational entropy.

## 1. Introduction

The 3rd industrial revolution started the digital era which began in the late 1950s–1970s with the introduction of digital technology and the movement away from mechanical and analogue technology. The digital revolution marked the start of the information age Schoenherr [1]. The 4th industrial revolution is marked by technological innovation in areas such as robotics, artificial intelligence, quantum computing, and biotechnology. Schwab [2] indicates that these new technologies are transforming industrial systems of production, management and employee qualitative and quantitative workloads.

Digitalization and automation introduced by the 4th industrial revolution has momentous and widespread effects on the workplace and employees. Like globalization there have been both benefits and costs in the digitalization process. On the negative side there has been the creation of a poorly paid underclass of workers forced out of skilled work by the introduction of automated and robotic processes in the workplace. The effects have been many on the incidence of voluntary and involuntary turnover, unemployment and well-being of the workforce. Desperation in the work force of the loss of traditional work opportunities and the threat to livelihood from digitalization and globalization have contributed to a wave of rebellion in the United States and ushered in a new era of politics focused on job protectionism and trade tariffs.

At the organizational level, employees have sought to make themselves less dispensable in the face of the wave of automation. This has led to extreme forms of worker organizational citizenship-type behavior in some situations through workers’ attempts to demonstrate their special worth to the organization. Through altruistic behavior and overt demonstrations of ‘going the extra mile’ for the organization, employees have sought to reduce their chances of redundancy. The effects have been many as Litchfield and Cooper [3] put it: “It is clear that work can be harmful but so can the absence of work. The link between poverty and illness has been recognized for many centuries but it was only in the 1930s that the independent effect on health of unemployment was first described. Research since that time has confirmed that both job loss and continuing ‘worklessness’ impact adversely on people’s health with increased levels of both mental and physical problems. Rates of anxiety, depression, suicide, hypertension, diabetes, stroke and heart attack have all been shown to be elevated in those who are made unemployed”. The pressure to keep employment in the face of automation and rapid industrial change in the 4th industrial revolution, has also had negative effects on maintaining a healthy work/life balance and forces workers to maintain their citizenship behavior far beyond formal office hours, thus introducing forms of citizenship extremism. As Harrison and Lucassen [4] indicate ”while in the past there was often a clear boundary between where work ended and home life began…this area is very much grey. Most of us have our work emails on our phones making us constantly available and contactable. This makes it very difficult to ever truly disengage from work and relax.”

Effects on well-being and mental health of the work force have been considerable, “For individual companies, mental health is now often the commonest cause of sickness absence in developed countries, accounting for up to 40% of time lost with presenteeism adding at least 1.5 to the cost of absenteeism” Litchfield and Cooper, [3].

Past and current secondary data evidence of extreme forms of organizational citizenship behavior as a reaction to highly stressful situations, suggest that extreme citizenship behavior can generate a ‘toxic’ spread among employees and the organization, ultimately leading to entropy. This paper aims to contribute to the literature by presenting a theoretical explanatory model of extreme forms of organizational citizenship behavior generated by highly stressed situations, experienced by employees in the digital era, which can generate disorder and entropy in business organizations.

The paper aims to show using a theoretical explanatory model how:

- Toxic forms of *leadership* brought on by pressures of rapid industrial change, automation, and information explosion of the 4th industrial revolution, and
- Pressures of the 4th industrial revolution in the form of redundancies, erosion of healthy work/life balances, and problems of mental health, have generated *toxic employee citizenship behavior* and threaten organizational entropy.

The paper takes the following structure: The first section presents a brief literature review of organizational citizenship behavior (OCB), entropy, entropic citizenship behavior (ECB), employee and organizational toxicity, and organizational entropy. The second section deals with methodology of secondary data analysis. This is followed by a presentation of a sequential model built up from the findings and analysis of the secondary data, and a discussion of employee and organizational toxicity and entropy in highly stressed 4th industrial revolution workplace situations.

The conclusion outlines limitations of the study, recommendations for further research and the paper’s implications for management in maintaining employee well-being and organizational sustainability.

## 2. Literature Review

This review describes the extant literature regarding various conceptual and theoretical foundations of the explanatory model developed later in the paper.

Organizational citizenship behavior (OCB) has interested social scientists for several decades [5]. Although OCB emerges as a distinct area of study, it has strong conceptual convergence with concepts such as: extra-role behavior [6], pro-social organizational behavior [7,8,9,10,11], and contextual performance [12,13,14]. Organ [15] originally defines OCB as: “individual behavior that is discretionary, not directly or explicitly recognized by the formal reward system”. More recently OCB is defined by Organ [16] as, “contributions to the maintenance and enhancement of the social and psychological context that supports task performance (or the technical/technological/production system”. Organ’s [16] more recent definition of OCB emphasizes its social and group level effects in the organization which underlines the core importance of OCB in organizational effectiveness 

Organizational citizenship behavior (OCB) has generally been associated with positive aspects of organizational functioning through its effect on the attainment of formal organizational goals. However, more recent studies have shown that excessive organizational citizenship behavior of “personal support” [17] and “organizational support” [18,19] types may be inimical to organizational goal attainment. Extreme personally supportive OCB may become detrimental to the accomplishment of organizationally proscribed tasks through, for example, spending large amounts of time helping coworkers in the workplace or performing extra-role socio-environmentally oriented tasks driven by toxic leaders, which negatively affect worker morale and productivity [19,20]. On the other hand, extreme organizationally supportive OCB may also have negative effects on the organization; for example, disruption caused by workplace norms and productivity through rate-busting [21] and burnout [18]. 

Having briefly outlined the concept of organizational citizenship behavior, it is necessary to discuss the origins of the concept of entropy in physical sciences and its adoption in the social sciences. The ‘entropy’ concept originates from the Greek en+tropein meaning “transformation content”. It is considered by Clausius [22] as that fraction of energy contained in a system unavailable to produce work; and that in any system this unavailable energy tends to increase. Landsberg [23], proposes a simple order/disorder entropy theory based on thermodynamics and information theory which defines entropy (total disorder) in a system that arises when a system’s capacity for disorder is ‘overwhelmed’ by its capacity for absorbing further information.

Theil [24], conceptualizes entropy as a measure of dividedness and dispersion in the development of his “evenness of spread” entropy concept. 

The social sciences have used the concept of entropy in a number of disciplines. For example, Bailey [25], working in the disciplinary field of sociology, maintains that “order is not a constant value but a matter of degree. Order can vary from zero (absolute randomness or maximum entropy) to one, or perfect predictability (maximum departure from randomness or minimum entropy)”. In zero order (maximum entropy) social systems, there will be maximum energy wastages. On the other hand, in social systems with minimum departures from randomness, there will be minimum energy wastages.

The notion of energy wastages in social systems is a common theme in various disciplines in the social sciences. For example, Gunn [26] defines business thermodynamics as energy transformation in a productive system which he regards as deriving its core energy from human motivation. Corporate entropy is regarded as that portion of a system’s energy that is unable to be transformed into a productive and functional system, and thus is irreversibly lost to that system. Ackoff [27] regards corporate entropy as being reduced through the elimination of wastage of corporate energy. If, for example, an executive manager uses unutilized board meeting time in motivating subordinates to increase work outputs, he/she will have reduced time wastage and corporate entropy by doing so.

In contrast to Theil [24], DeMarco and Lister [28] define corporate entropy as “levelness or sameness”. The more ‘sameness’ increases, the more the potential to create energy to do work diminishes. Uniformity in attitudes and thought processes in a corporation is regarded as entropy because they tend to smother productive work energy. DeMarco and Lister [28] maintain that organizational entropy is engendered by increases in ‘staleness’. General organizational stasis is often found in older corporations with tightly structured bureaucracies. 

Williams [29] defines organizational entropy as the *disorder* in the organization and in the performance of work functions. For example, if person A is working on project Y and needs person B’s skills to accomplish a task but, person B is already working on project X, project Y is held up. 

DeMarco and Lister [28] suggest that in large organizations with multiple projects occurring simultaneously, disorder in energy utilization leads to energy wastage and entropy. They indicate that entropy arising from such sources could be counteracted by managerial interventions, such as critical path analyses. 

The selected examples described above clearly show that organizational forms of entropy are associated with the common underlying dimension of *energy wastages* leading to disorder and entropy in social systems. This idea is now developed further with the concept of OCE in the literature review.

Organ’s [15], definition of OCB as a phenomenon that extends beyond individual to group level has multiple implications for the importance of OCB in organizational entropy. Two major aspects of Organ’s [15] definition of OCB emerge the; conception of “individual” “conscientiousness” and the conception of “group morale”. From this it seems reasonable to suggest, although not specifically indicated by Organ [15], that *group morale* arises largely from the collective aspects of *individual conscientiousness* being ‘joined together’ and is considered an important aspect of OCB. 

At the individual level of analysis, OCB is usually regarded as multifaceted and incorporating aspects of altruism, compliance, sportsmanship, courtesy, and civic virtue. In general terms, however, OCB can be, as it is in the current paper, conceptualized as being a basic dichotomy made up of *organizational and personal support* dimensions [16]. Findings of empirical studies of OCB [19,29], suggest that *balance* between personal and organizational goals in organizational citizenship is critical for good organizational performance and that extremes of either *personal* or *organizational* citizenship orientations, may increase organizational entropy propensities. Theoretically it is maintained, in line with Coldwell’s model [30], that increasing amounts of either personally or organizationally oriented OCB, distributed unequally amongst categories of organizational personnel, will result in disorder and increases in organizational entropy.

In the original model proposed by Coldwell and Callaghan [31], organizational citizenship entropy (OCE) is defined as an extreme form of organizational citizenship behavior that occurs at very high levels of personally oriented citizenship behavior and very low levels of organizationally oriented citizenship behavior, and vice versa, Historical examples from the extreme contextual circumstances created by war are used as secondary empirical data to validate the heuristic of OCE developed by Coldwell and Callaghan [31].

Very briefly. Axelrod’s [32] insight into extreme personal oriented citizenship behavior demonstrated in the altruism of front line trench soldiers in the First World War indicates that the military’s formal organizational goals of applying lethal force involved both killing and the prospect of being killed, could be undermined by interpersonal supportive behaviors of “live and let live” adopted by soldiers on both sides in the opening stages of the war. Ultimately, the effect of this behavior would have been to undermine the purpose of waging war which is to ensure victory and the destruction of the enemy, had it been allowed to persist. 

The highly stressed contextual circumstances of warfare have also been shown to generate extreme forms of organizationally oriented citizenship behavior leading to OCE. Coldwell and Callaghan’s [31] original heuristic did not formally present the kind of disordered distribution of personal and organizational citizenship behavior that led to OCE. This aspect was taken up by Coldwell [30]. who uses Theil’s [24], “evenness of spread” concept to provide a more formal and detailed development of the original model Theil’s [24], conceptualization of entropy emphasizes “evenness of spread”, Theil [24], considers the proportion of the maximum possible dispersion in which a variable is spread among categories or spatial units. This is 1, if the variable is evenly spread among all categories or 0, if the variable is concentrated in a small number of categories. “Categories” in the current paper, refer to divisional and departmental personnel. When specific types of personal or organizational oriented citizenship behavior are concentrated among individuals in units, teams and departments, and unevenly those units, teams and departments tend towards entropy through the toxic effect of this behavior on other employees. 

Coldwell’s [31] formal model of organizational citizenship entropy consists of an inverted U-shaped curve with *organizational sustainability* on the vertical axis and *levels of personal and organizational-oriented citizenship* behavior on the horizontal axis.

The relative evenness and unevenness of the OCB distribution is seen as ranging from 0 (complete uniformity) to H (complete chaos). Points beyond H Min (minimum level of disorder) become increasingly even in OCB as they progress towards point 0 (complete uniformity). Beyond point H Max, (maximum level of disorder) increasingly uneven distributions of personal and organizational OCB become evident. Organizationally- and personally oriented OCB between H Max and H Min are regarded as areas of order in organizational departments (an evenness of OCB distribution without extreme forms). In this area, balanced forms of OCB become increasingly evident. Increasing imbalances (extremes) in personal and organizational OCB occur towards H Max and, become OCE. OCE in units and teams ‘poisons’ the organizational climate and can spread to other departments resulting, ultimately, in the development of a toxic organization.

In general terms, Coldwell’s [31] model suggests that an absence of extreme manifestations of personal and organizational OCB distribution in units, departments and divisions, promotes organizational sustainability and reduces the probability of toxic employees’ OCE spreading and generating a toxic organization, but complete evenness in OCB may generate stasis and rigidity making the organization less able to adapt to rapid industrial change [16].

Appelbaum and Roy-Girard [33] state: “Toxicity is a fact of life in all organizations; however, not all organizations are toxic. Toxic organizations are usually defined as largely ineffective as well as destructive to its employees. Simply having toxins present in an organization does not necessarily make it a toxic organization. The tone of an organization tends to be set from the top and so toxicity is often a top-down phenomenon. The higher up the toxic person is, the more widely spread is the pain, and the more people there are who behave in the same way”. This definition indicates that toxicity, particularly when originating at leadership levels, generates a poisonous climate in an organization. The use of the concepts of toxin and toxic to describe the destructive, spreading aspect of poison introduced to a system is considered particularly apt in relation to organizational entropy because of the instability (disorder) toxins are known to generate in a system. Although, as Applebaum and Roy-Girard [33] suggest, toxins in an organization are often introduced by a leader and spread through the organizational climate created by that leader to managers and other employees, it is unlikely that a leader, however forceful or pervasive his/her influence might be, can create a toxic organization on his/her own. She can, however, create a climate conducive to its spread and propagation.

Toxic leadership can therefore be regarded as a necessary, but not necessarily a ‘sufficient’ condition for the emergence of toxic organizations. A toxic organization can be initiated through leadership behavior, but this can only be spread throughout the organization through the collective interaction of toxic employees. Toxic leaders do not just materialize out of the air, they are usually the result of socio-economic pressures, such as those created by the 4th industrial revolution with its emphasis both on competitive efficiency through technology and automation, and environmental sustainability. 

Rasool et al. [34] indicate how workplace toxicity can negatively influence productivity through the mediating role of depression: which leads “…employees to undermine performance and leave a bad image of the workplace among their peers”. Rasool and Summa [34] conclude that “…a toxic workplace environment increases the level of work depression. When workers feel negative about their organization, they tend to compromise their productivity level, which could also increase their level of trauma.” Moreover, as Anjum et al. [35] note, a toxic work environment negatively impacts on job productivity and generates employee burnout.

Toxic employees and organizations can arise from many sources but distorted types of leadership which drive distorted employee behavior and a toxic organizational climate are becoming more prevalent in the digital era. Toxic employees and organizations arise from contextual stressors created through extreme circumstances such as economic and socio-political turmoil arising from, for example, rapid industrial change (revolution) and war.

## 3. Methodology

The paper uses secondary data analysis of extant literature to build and consider the utility of an organizational citizenship theoretical model in explaining toxic forms of employee behavior that lead to toxic organizations and organizational entropy. Secondary data analysis is: “In the broadest sense, (an) analysis of data collected by someone else” [36]. An analysis of secondary data involves the use of secondary information obtained from existing sources and, “…can include any data that are examined to answer a research question other than the question(s) for which the data were initially collected” [37]. The approach is distinct from primary data analysis which involves the same individual(s) designing the research, collecting the data, and performing the analyses. Novel findings can usually only be generated from primary data sources. 

Cooper and Schindler [38] indicate internal and external categories of secondary data and three types of sources of this material namely; primary, secondary, and tertiary. Primary sources are original raw data before interpretation. Secondary sources are original works of research which have been interpreted. Examples of tertiary sources are interpretations of secondary data, indices, bibliographies and Internet research engines. Despite the explosion in the volume of secondary data available to the researcher today, secondary data analysis remains an underutilized resource. In some instances, such as in the current paper, few other readily available data resources are available that can provide the researcher with the empirical means to construct theoretical models. However, secondary data sources need to be scrutinized for their reliability and validity. Reliability refers to the consistency of the data obtained and is assessed in the context of the current study, by the consistency of different reports of specific company situations. Validity concerns the credibility of the secondary data. In the context of the current study, it seeks to interrogate whether the data shows what it purports to show by assessing the credibility of the sources producing it. Such data can be subjected to the scientific criterion of falsification through an analysis and interpretation of extant secondary data assessing its goodness of fit with a proposed heuristic and its degree of corroboration over time. However, such heuristic devices built up from interpretations of secondary data remain tentative and open to change. Using Popper’s [39] celebrated analogy, the fact that all swans I have seen so far are white and I have never seen a black swan, does not mean that all swans are white.

Secondary data analysis can be used, as it is in the current paper, to provide empirical support for theoretical heuristic models [36], but the model remains tentative and open to falsification. 

The secondary data in the current paper was obtained from the collection of data from reliable and credible external sources of reports of business organizational crises in which the author did not participate and had no influence on their specific research designs, or the research questions they sought to investigate [36]. These aspects of research non-involvement are specific disadvantages of secondary data analyses.

## 4. Findings of the Secondary Data Analysis

### 4.1. Toxic Organization-Oriented Citizenship Behavior

In the original model put forward by Coldwell and Callaghan [31], historical secondary data describing the behavior of combatants in the First and Second World Wars was used to support a heuristic that suggested that the extreme circumstances of war led to extreme forms of personal and organizational citizenship behavior which were inimical to organizational effectiveness. Very briefly, in the First World War personally oriented citizenship behavior between the British and German entrenched soldiers led to both sides pretending to wage war which allowed both sides to avoid casualties altogether. This extreme personally oriented, “live and let live” citizenship behavior was inimical to the goals of the military organization [32]. In the Second World War, extreme organizational oriented behavior which was ultimately inimical to the Japanese war effort was displayed by Japanese kamikaze pilots. [40] 

The concept of compulsory citizenship behavior (CCB) also has specific relevance in this context. Vigoda-Gadot [18] describes CCB as:

“…behavior that, in contrast with conventional OCB, is not based on the genuine, spontaneous “good will” of the individual. Instead, it emerges in response to external pressures by significant and powerful others in the workplace (i.e., managers or co-workers) who wish to increase the employees’ workload by involving them in duties that are beyond the scope of their job description” [18].

Moving from the extreme circumstances of war to the extremes of rapid industrial change in the digital era, an empirical example of toxic organizational citizenship behavior in a business organization is provided by the recent Volkswagen crisis. A group of company engineers decided to distort diesel emissions tests on its cars because a technical solution to the problem could not be found in the time made available to do this and within the company’s budget. Analysis of the Volkswagen crisis shows that fertile grounds for this toxic form of employee behavior were laid through the influence of toxic leadership. Driven by the company’s determination to succeed in becoming the world’s top selling car maker, company leaders were prepared to create an organizational climate that would stop at nothing to attain this goal. The company was run by a highly centralized authoritarian hierarchy in Wolfsburg that expected employees to ‘deliver the goods’ no matter what, which put enormous pressure on them to achieve organizational goals and to work 24/7 until a solution had been found. Such toxic leadership focus generated toxic employee behavior that became culturally embedded in the VW organizational climate [41]. Although VW survived the ’Dieselgate’ crisis, it cost the company in the region of $30 billion USD to resolve [42].

The potential of VW leaders and employee toxic behavior spreading to other organizations and affecting their production and to the German economy is clearly described in an article [43] which indicated that the: “recent emissions scandal is most likely to have far-reaching consequences. Rigging pollution results will not only cost the automaker dearly in terms of legal fines, investor and customer backlash, class action suits, possible criminal investigation, and loss of future sales, but the ill-effects of this scandal could spill over to other automakers, particularly Germans who make cars that run on diesel, and have a broader impact on the automotive industry. In fact, given how this scandal has everybody raising their eyebrows at the previously trusted and respected German engineering, the blow to the country’s largest automotive company could, in turn, hurt the country’s economic growth”. The effects of widespread access to information brought into being by the technology of the digital era, have made the VW problem, accessible to stakeholders and the general public heightening its impact and spread. Broadly speaking, the toxic employee behavior being encouraged by the VW leadership hierarchy can be regarded as empirical examples of extreme forms of *organization-oriented OCB*, where employees were driven to go the extra mile for the company and forced to attain company goals no matter what. This extreme form of company-oriented OCB, in turn, generated OCE at VW which and impacted on the automotive industry as a whole and spread to other sections of German industry dependent on the automotive industry for economic sustainability. 

A second more recent example of organizational-oriented citizenship behavior in extreme form can be obtained from the crisis at Boeing. The recent crashes of the 737 Max aircraft, with complete loss of life of passengers and crew in both cases, presents further example where the leadership of a large organization with a highly reputable history, turned toxic from pressures of industrial competition brought about by relentless advancements in aeronautical automation and the company’s drive for profits. The pressure from competition came from Airbus and the problem Boeing confronted was to produce an aircraft that was 15% more fuel efficient in time to successfully compete with Airbus. Leadership was under pressure to produce a solution in a short time frame and this was filtered through to the engineers and computer personnel who were beset with a target ‘to do whatever was necessary’ to meet the objective set in the limited time available. As with the VW crisis, the toxic leadership pressure generated organizational-oriented citizenship behavior ‘to go the extra mile’. Among individual employees the pressure was to find a solution in a short span of time generated 24/7 burnout and created a toxic employee environment. Toxic leadership pressure also affected the Federal Aviation Administration (FAA) whose job it is to license aircraft only when they met strict safety requirements. The FAA duly gave its safety approval for the 737 Max despite its obvious faults. As Pontefract [44] puts it, “The FAA with its reduced staff and growing lists of actions to take care of might have simply been fine with it. Maybe it even freed up time to get other actions completed on their lists. Boeing—in a herculean race to keep pace with Airbus—were likely keen to ensure that the plane made it to the market as soon as possible, and with the least amount of disruption and cost overruns (let alone pilot training cost concerns from the airlines)”. The rushed-out solution was the Maneuvering Characteristics Augmentation System (MCAS), which was a computer-automated stall-control system that overrode pilot control of the ‘angle of attack’ when it deemed, from information obtained from electronic sensors, that a stall was imminent. Entropy and disorder at Boeing through its energy-wasting attempts to install an automated system to help fly an aircraft designed more than 50 years ago, without a fundamental aeronautical change is already evident, but the threat to Boeing’s survival, while severe, has yet to fully unravel. In this regard a crucial additional aspect to Boeing’s long-term survival which is often overlooked but may be more important than the purely technical one, is consumer reaction. Will consumers simply refuse to fly in a modified Boeing 737 Max in the future? Future consumer reaction to the modified Boeing 737 Max has yet to be witnessed [45]. 

### 4.2. Toxic Person-Oriented Citizenship Behavior

Paillé and Grima [46], in a paper focusing on organizational citizenship withdrawal in the workplace, found that an emphasis on helping others (personal-oriented citizenship behavior) was a strong predictor of employee intention to leave. This finding suggests that digital era pressures on employees to engage in personal/helping citizenship behavior produced toxic reactions that caused them to consider quitting the workplace. Such individual employee workplace withdrawal becomes dangerous to organizational survival when it diffuses to create a toxic organizational climate. An example of this phenomenon with a more widespread impact is presented by Roddick’s Body Shop [47]. The Body Shop was founded on social activism and the principles of social and environmental change. Anita Roddick saw business as not simply the unwavering pursuit of profit to build a larger commercial empire, but also to influence social change and to make the world a better place to live in. Her focus was primarily on human rights and environmental preservation and she surrounded herself with employees who had been carefully selected to embrace these values. However, her company leadership ultimately created a toxic climate where the drive and emphasis on people and community welfare generated employee social activism that became inimical to the economic sustainability of the company itself. Open access of information about the company made possible by digital technology made the company become the object of a number of ethics controversies and was eventually sold to L’Oréal. The company is now owned by Brazilian Cosmetics Company "Natura Cosméticos" who bought the company from L’Oreal in 2017.

Murray’s [48] account of Ben and Jerry and CPI’s struggles with corporate social responsibility provides a more recent example of how extreme forms of personal-oriented, ‘helping’ organizational citizenship behavior brought about by pressures for social responsibility in the digital era, can create toxic organizational circumstances which threaten its existence. In this particular case, Ben Cohen, a co-founder of Ben and Jerry, after attending a concert by the Grateful Dead to promote the protection of the Brazilian rainforest, embarked on a partnership venture with Community Products Inc., (CPI) to produce a new ice cream,’ Rainforest crunch’ made from Brazilian nuts and cashews. Ben Cohen, who was chairman and president of CPI, committed the company to distribute 40% of the profits to rainforest preservation groups and international environmental projects. Another 20% would go to 1% for peace, and a further 10% would be shared among employees. CPI’s social and environmental claims eventually came under scrutiny by information available to stakeholders and the general public. The original small cooperative nut farmers could not meet with the increasing demand for the product and comply with US health standards. Shipments of nuts arrived with broken shells, cigarette butts, rocks and coliform bacteria. It was found out also that 95% of the nuts were supplied by large corporate suppliers, including notorious anti-union agribusinesses. In fact, only 5% of the nuts came from local co-operatives. Ben and Jerry were forced to backtrack on their social responsibility claims and Rainforest Crunch was discontinued and CPI went bankrupt leaving its employees redundant and with some small local cooperative nut suppliers remaining unpaid. 

In such circumstances the organization had become so fixated on its helping solve community social and environmental problems citizenship behavior, that it was prepared to go bankrupt (entropy) in its drive to maintain its reputation in the face of negative information, despite its erstwhile company profitability. Such toxic leadership puts enormous pressures of possible redundancy on employees which can create a toxic organizational environment to emerge with the twin objectives of maintaining a community-oriented façade while struggling to remain in business. 

## 5. Discussion of the Findings and the Development of a Model

The secondary data indicated in the preceding section has suggested that extreme forms of personal and organizational-oriented citizenship behavior can, under the pressures of the 4th industrial revolution and toxic leadership, lead to organizational entropy.

**Figure 1:**
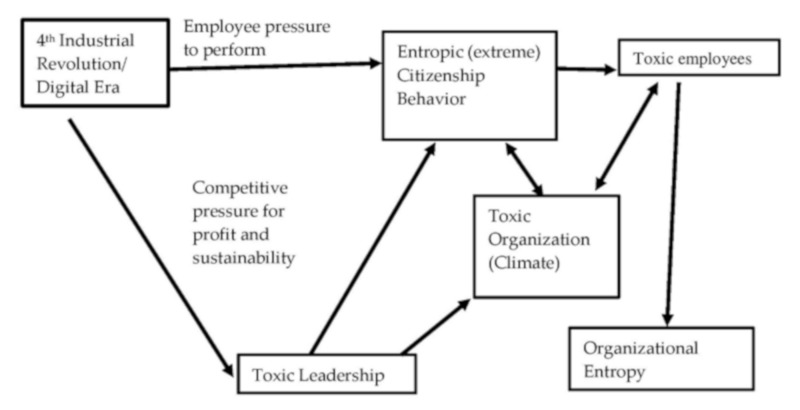
A theoretical model of negative influences of the fourth industrial revolution on workplace citizenship behavior.

Such compulsory extreme forms of citizenship behavior [18] engendered by pressures of the 4th industrial revolution have been shown in the examples described earlier to seriously undermine the functioning and survival of organizations. Figure 1 below presents, diagrammatically, a heuristic of evident links in the chain of organizational citizenship entropy observed in the secondary data presented earlier. 

Figure 1 indicates that pressures in inter-firm competition prompted by the rapid changes of the 4th industrial revolution has promoted toxic forms of leadership in organizations. Pressures on employment from automation and robotics as well as the intrusive technology that has arisen in the digital era have also driven individual employees to work 24/7 and to try to demonstrate in extreme forms of personal and organizational-oriented citizenship behavior, they are worthy of continued employment. These twin sources of pressure encouraged by toxic leadership have driven individual employees towards OCE through burnout and withdrawal which have helped create the toxic organizational climate and which they are themselves affected by (depicted by two-way arrows in Figure 1). Energy wastages and disorder created by ECB can ultimately lead to entropy of the organization itself. The model indicated in Figure 1 goes further than Einarsen et al.’s [49] earlier descriptive model of ‘destructive leadership’. Destructive leadership is defined by Einarson et al. [49] as systematic and repeated behavior by a leader to undermine or sabotage the organization’s goals and the well-bring and job satisfaction of employees. The definition recognizes that destructive leaders can display destructive and constructive behavior simultaneously, however, their descriptive model [49] does not present an explanation of *how* destructive leadership tendencies might be developed. Additinally, Einarsen et al.’s model [49] does not consider how destructive leaders might arise from the relentless drive for what might at first sight appear to be perfectly legitimate goals aimed at contributing to organizational and/or socio-environmental objectives. However, the secondary data analysis has indicated how fixated organizational and environmental goals strongly motivated in company employees by toxic leaders can lead to organizational crises and even catastrophe. 

## 6. Conclusions

This paper has indicated through secondary data analysis and the development of a novel theoretical modular framework, interrelationships between 4th revolution pressures that promote leadership toxicity, employee entropic citizenship behavior, employee and organizational toxicity and, ultimately, organizational entropy. The paper contributes to the literature by presenting a theoretical framework built from extant secondary data that articulates testable relationships for future primary data empirical research. It has also shown how pressures emanating from, but not necessarily unique to, the 4th industrial revolution have impacted on the workplace and, in some cases, generated extreme forms of personal and organizational-oriented citizenship behavior that lead to energy wastages, disorder, and organizational entropy.

The model suggests that management and stakeholders need to be alert to toxic leadership and to avoid recruiting company leaders who show toxic tendencies in their overriding fixations on community/environmental or profitability goals often arising through 4th industrial revolution pressures. The model also alerts management to possible digital era pressures on employees that drive them, sometimes through threats of redundancy and unemployment, to ‘go the extra mile’ for the company by extreme forms of citizenship behavior. The model shows how extreme forms of employee citizenship behavior can emerge from toxic leadership pressure and/or the erosion of job security and work/life balances associated with the digital era. From a practical point of view, instances of burnout, depression, and general mental illness need to be carefully monitored by management. Prescriptive remedial steps need to be taken by management through the implementation of specifically designed training programs to alleviate stress. Management needs to be alert to identifying, reporting, and responding to toxic behavior [35] and, when considered necessary, the active intervention of mental health providers. 

The paper is limited by the fact that it is *conceptual* and further testing of the modular framework presented using primary research data is called for before the model can be considered generalizable. In this regard it is recommended that further primary research should use qualitative and quantitative analyses to establish the validity and generalizability of the model. The paper has also depended on secondary data reports that are the perceptions and interpretations of second party investigators which have then been further interpreted by the current researcher. As mentioned earlier this tertiary data interpretation remains tentative and open to change. The paper has focused on negative influences of toxic leadership and extreme forms of citizenship behavior that can disrupt a company’s performance and threaten its survival. This can occur, as we have seen in the secondary data discussed in the paper, but in many cases, it occurs through breakdowns in organizational checks and balances. As Chen and Beers [50] point out “By separating the duties of various employees into clearly defined roles, businesses, and organizations are better able to ensure that rogue employees or executives cannot harm a business without the intervention of other employees. Having these types of internal controls in a business can help improve operational efficiency”. However, it is nevertheless true that effective checks and balances are more prevalent in the government than private sector where rogue forms of leadership and employee behavior are more evident.

The paper has suggested that there are twin sources of stress in the workplace generated by unique pressures created by the rapidity of change in the 4th industrial revolution. The first source of pressure towards extreme forms of citizenship behavior is intrinsic to the employee. The digital era has created technology that allows no respite for employees from the responsibilities of the workplace; they, in effect, continue working in the office even when away from the office, 24/7. This has created extreme forms of compulsory-type citizenship behavior [18], aided and abetted by the constant threat of redundancy which has led inexorably to increases in problems of employee well-being and mental health evidenced in cases of withdrawals from the workplace and withdrawals from work while being at work (presenteeism) and complete burnout. The second source of extreme forms of citizenship behavior emerging in the workplace has been, the paper suggests, from toxic leadership, themselves driven by the competitive pressures of the 4th industrial revolution of automation, efficiency, and corporate social responsibility. These two primary sources of digital era pressure create fertile ground for extreme forms of citizenship behavior, disorder, and entropy.
